# Do individualism and collectivism on three levels (country, individual, and situation) influence theory-of-mind efficiency? A cross-country study

**DOI:** 10.1371/journal.pone.0183011

**Published:** 2017-08-23

**Authors:** Tuong-Van Vu, Catrin Finkenauer, Mariette Huizinga, Sheida Novin, Lydia Krabbendam

**Affiliations:** 1 Faculty of Behavioural & Movement Sciences, Vrije Universiteit Amsterdam, Amsterdam, the Netherlands; 2 Department of Interdisciplinary Social Sciences, Utrecht University, Utrecht, the Netherlands; 3 Department of Social, Health, and Organizational Psychology, Utrecht University, Utrecht, the Netherlands; University of Stirling, UNITED KINGDOM

## Abstract

This study investigated whether individualism and collectivism (IC) at country, individual, and situational level influence how quickly and accurately people can infer mental states (i.e. theory of mind, or ToM), indexed by accuracy and reaction time in a ToM task. We hypothesized that collectivism (having an interdependent self and valuing group concerns), compared to individualism (having an independent self and valuing personal concerns), is associated with greater accuracy and speed in recognizing and understanding the thoughts and feelings of others. Students (*N* = 207) from individualism-representative (the Netherlands) and collectivism-representative (Vietnam) countries (Country IC) answered an individualism-collectivism questionnaire (Individual IC) and were randomly assigned to an individualism-primed, collectivism-primed, or no-prime task (Situational IC) before performing a ToM task. The data showed vast differences between the Dutch and Vietnamese groups that might not be attributable to experimental manipulation. Therefore, we analyzed the data for the groups separately and found that Individual IC did not predict ToM accuracy or reaction time performance. Regarding Situational IC, when primed with individualism, the accuracy performance of Vietnamese participants in affective ToM trials decreased compared to when primed with collectivism and when no prime was used. However, an interesting pattern emerged: Dutch participants were least accurate in affective ToM trials, while Vietnamese participants were quickest in affective ToM trials. Our research also highlights a dilemma faced by cross-cultural researchers who use hard-to-reach populations but face the challenge of disentangling experimental effects from biases that might emerge due to an interaction between cultural differences and experimental settings. We propose suggestions for overcoming such challenges.

## Introduction

Making inferences about other people’s unobservable thoughts and feelings is an essential skill required by human beings to navigate social life, which is usually referred to as theory of mind (ToM) [1]. To be able to mentalize or to have ToM is thought to enable human social processes such as empathy, sympathy, and compassion [2,3]. ToM also facilitates social interactions such as prosocial behaviour [4], moral judgments [5], and conflict resolution [6]. Given its significance, there is a keen interest across fields of psychology in the factors contributing to effective ToM [7,8]. Previous research suggests that ToM is influenced by *individualism* and *collectivism* (IC) [9]–the cultural orientations defined by whether more emphasis is placed on the individual or on the group–which are usually attributed to cultural differences between countries (e.g., China vs the United States) [10]. Collectivism-representing Chinese participants have been found to have more accurate ToM and ToM-related performances than their individualism-representing American counterparts [9]. However, recent advances in cross-cultural psychology have demonstrated that variations in IC not only occur across countries but also across individuals [11,12] and across immediate situations [13,14]. Whether and how these variations on the different levels might have an effect on how one makes inferences about the thoughts and emotions of others is a question that is currently unanswered. In the present study, we aim to systematically examine the possible effect of each of these three levels of IC (the country, individual, and situational levels) and their interactions on the efficiency with which individuals can infer mental states of others. ToM efficiency is indexed by ToM accuracy and speed.

### ToM as ability and as propensity

ToM is most often defined as the ability to recognize mental states, including the diverse thoughts, intentions, beliefs, emotions, desires, and wishes of other people, and to accurately make meaning of these mental states [15,16]. Despite relative consensus on its definition, ToM is notoriously difficult to assess, and researchers typically use a wide range of tasks that tap into different aspects of ToM in various experimental settings. Child ToM research most often uses false belief tests, which assess whether a child (usually younger than 4–5 years old) can understand that another person might hold a belief that is not in line with reality and that is different from the child’s (e.g., the Sally and Anne test [17]). The result of this test is widely considered a hallmark of whether a child has developed a conceptual ToM.

In healthy older children and adults, who are assumed to have already established a conceptual understanding of false belief [18], other ToM approximations, such as perspective-taking, egocentric bias, empathic concern, and empathic accuracy, are studied. Perspective-taking is the ability to represent or to adopt the view of another or “the ability to put oneself in the shoes of another” [19]. In a typical perspective-taking task, participants are instructed to relocate objects in an array by another person who can only see a subset of objects that the participant sees because he or she stands behind the array. To move the correct object, participants need to adopt the viewpoint of the other person. Egocentric bias is the degree to which one overestimates how similar their thoughts and feelings are to those of another person, usually because one uses the self as the anchor [20]. It is usually measured by asking participants to describe what a target person feels. Empathic concern, the degree to which one has an other-oriented emotional response [21], is usually measured with self-reports. ToM is also sometimes approximated by measuring empathic accuracy, which is how accurately one can describe another person’s feelings [22]. Finally, there are also a number of ToM tasks that measure how one infers both the thoughts (i.e. cognitive ToM) and feelings (i.e. affective ToM) of others, with the aim of tapping into a broad range of aspects of ToM (e.g. the cartoon vignette ToM task [23]).

Early accounts of adult ToM assumed that healthy adults make perfect use of ToM because they pass the false belief task [7,24]. However, research assessing perspective-taking, egocentric bias, empathic concern, and empathic accuracy has consistently found that, although adults have perfect conceptual ToM, they do not always make accurate and timely inferences about the thoughts and feelings of others [24–26]. Instead, external demands (e.g., in-group vs out-group context), individual differences in responding to external demands (e.g., the capacity for social information), and the relationship to ToM target (e.g., being a close other or being in a position of greater power) influence the accuracy and speed of ToM processes. For example, the deployment and speed of ToM processing both decrease in the presence of out-group members, compared to in-group members [27–29]. People who have a larger social network are able to keep better track of what is on other people’s minds than those with fewer close others [30]. Participants who were experimentally assigned to a position of greater power were worse at understanding participants in a position of lower power, while participants in a position of lower power were better at understanding those in the position of greater power [31]. These results suggest that there is more to ToM than ability alone.

These findings are in line with recent advances in ToM research, which contend that ToM variations should not *only* be assessed as differences in ability (i.e. the current potential under optimal conditions). Rather, ToM variations between people should be evaluated as an effect of propensity (i.e. the expression of ToM as a function of the condition) [32]. The success with which people make inferences about the thoughts and feelings of others includes the capacity to deploy their ToM in a timely and contextually appropriate manner [18]. In the present research, we are interested in the possible influence of culture on these variations in ToM.

### Individualism and collectivism (IC) and their consequences for ToM performance

It seems that different cultural orientations, among which individualism and collectivism (IC) figure prominently, can have an impact on people’s ToM. Collectivism–a cultural orientation that is predominant in East Asia–refers to assessing the self in relation to others (interdependent self-construal) and placing group concerns (e.g., group harmony and cohesion) above personal concerns (e.g., self-enhancement). In contrast, individualism–a cultural orientation that is predominant in Western Europe and North America–refers to assessing the self as separate from others (independent self-construal) and placing individual concerns above those of the group [33,34]. People from different cultural spheres may use both assessments of the self, but sample elements of these two selves with different probabilities [35].

Due to this essential difference, it is conceivable that collectivism is associated with other-oriented cognition, emotion, and motivation, while individualism is associated with self-oriented cognition, emotion, and motivation [34,36]. Indeed, researchers have found that people who have a predominantly interdependent self tend to take the perspective of another person, while those who have a predominantly independent self mentalize from their own egocentric point of view [37]. An interdependent self-construal might also be linked to other-oriented behavior. Ybarra and Trafimow [38] found that people with a stronger interdependent self were more likely to behave in line with what they believed others expected. For individuals with an independent self, internal desires were more influential in shaping their behaviors.

In summary, it seems plausible that collectivism is associated with other-oriented cognition, emotion, and motivation, while individualism is associated with self-oriented cognition, emotion, and motivation. As a result, we predict that collectivism, compared to individualism, might entail faster recognition and more accurate understanding of the thoughts and feelings of other people. The hypothesized superiority in ToM efficiency in individualism or collectivism does not imply better social functioning in an absolute sense, but rather increased ToM efficiency given the requirements of an I or C society, an I or C personality, or an I or C situation.

### The effects of country, individual, and situation IC on ToM

#### The effect of Country IC on ToM

Typically, cross-cultural studies compare individuals from North America or Western Europe (representing an individualistic culture) with those from Asia (representing a collectivistic culture). These studies provide some support for the suggestion that collectivism, in comparison to individualism, might be associated with more efficient ToM. To illustrate, Cohen and Gunz [39] found that Easterners/Asian Americans (representing a collectivistic culture) were less susceptible to egocentric bias than Westerners/European Americans (representing an individualistic culture). In a similar vein, Wu and Keysar [9] used the above-mentioned perspective-taking task and analyzed whether and how long participants’ eye gaze remained on the foil and target objects. They found that (individualism-representing) American participants made significantly more mistakes in perspective-taking than the (collectivism-representing) Chinese participants. More recently, Atkins et al. [22] also found that (collectivism-representing) Chinese participants were more accurate in judging someone else’s social pain (empathic accuracy), compared to (individualism-representing) British participants. On the basis of these findings, it appears that IC on the country level (which we label Country IC in the following) may have an impact on ToM: living in a predominantly collectivistic society, as opposed to a predominantly individualistic one, seems to entail the motivation, tendency, or ability to more quickly become attuned to and more accurately comprehend the thoughts and feelings of others. In the present research, we include Country IC as one level of analysis to examine how ToM efficiency varies across participants from individualistic versus collectivistic countries. Moreover, in the light of new advances in cross-cultural psychology [11,40], which advocate multilevel analyses of culture, we propose that IC may be reflected at two other levels, which might also affect people’s ToM efficiency.

#### The effect of Individual IC on ToM

Existing studies examining the effect of IC on ToM have based their conclusions solely on cross-country comparison and overlooked evidence suggesting that IC varies across individuals (which we label Individual IC). However, culture is not static, it constantly changes across time for every group and every member of each group [11,12]. Moreover, individuals across cultures may endorse IC values to different degrees [12]. When IC first came into the spotlight in cross-cultural psychological research, Triandis [11] argued that IC at the individual level is distinctive from that at the country level. In a recent meta-analysis, Taras et al. [41] found that IC measured at the country and the individual levels fit two distinctive dimensional structures, suggesting that IC effects are likely to vary depending on the levels of analysis. In summary, researchers have recognized that cross-cultural psychology needs to take into account Individual IC when examining cultural differences, over and above Country IC effects [12,42].

Although not yet incorporating two levels of IC, some previous research has investigated the sole effect of Individual IC on ToM. Duan, Wei, and Wang [43] found self-reported empathic concern to be correlated positively with collectivism and negatively with individualism on the individual level. Balcetis et al. ([44], study 4)also found that those with predominantly individualistic values, compared to those with predominantly collectivistic ones, made more erroneous predictions of the behavior of others, resulting from egocentric bias. These findings suggest that we should also expect that individuals who score high on collectivism, compared to those who score high on individualism, would have more accurate and faster ToM.

#### The effect of Situational IC on ToM

In addition to IC on an individual level, many existing studies have also overlooked the possibility that IC may vary at the situational level (which we label Situational IC). Culture as Situated Cognition (CSC) theory [14,45] posits that culture does not rigidly prescribe but rather provides situational cues for how people make sense of and organize their social world. Cross-country differences in IC do not emerge due to the presence or absence of IC but due to *chronic* contextual between countries. Individualistic and collectivistic mindsets are thus contextually constructed and include values, self-concept, cognitive styles, and goals that have a direct influence on cognition, emotion, and behavior [46]. Depending on the immediate context, different mindsets may be cued. For example, both an East Asian and a Western European person may adopt a primarily collective mindset in a collaborative situation (e.g., family gathering), but act with a primarily individualistic mindset in a competitive situation (e.g., university entrance exam). Empirical research has indeed found that some cultural differences are situation-dependent and malleable [11,47–49]. In the same vein, research within the CSC framework has also shown that cultural priming can make either collectivism or individualism salient for a situation at hand, which has a carry-over effect on a subsequent task [46].

Thus far, we are aware of only one study investigating the effect of a form of cultural priming on perspective-taking. Luk, Xiao, and Cheung [50] used cultural icons to prime participants with individualism (e.g., American Superman) or with collectivism (e.g., Chinese Confucius) and measured their performance in the perspective-taking task. They found that the participants who were in the individualism-primed condition were more likely to make perspective-taking mistakes–by moving the wrong object–than the participants in the collectivism-primed condition. In the present research, we took a further step by using cultural primes rather than country primes to ensure that what is primed are universal individualistic and collectivistic concepts, which can be made accessible in all people, instead of culture-specific aspects only accessible to a particular culture or country [51,52]. In addition, we aimed to complement previous research by using a comprehensive ToM task. We expected that those who were primed with collectivism (i.e. being placed in a situation that cues collectivism) would have more accurate and faster ToM than those primed with individualism (i.e. being placed in a situation that cues individualism).

#### The interplay between the three levels of IC

Since culture is an inherently multilevel construct, many researchers in the field have emphasized the importance of a multilevel approach to studying the effect of culture on cognition and behavior [40,53,54]. Nevertheless, despite considerable progress in the development of multilevel theories, few culture studies attempted to bridge these levels of analysis [54]. In addition, previous studies on the link between IC and ToM usually relied on a single level of IC and did not investigate the interplay between the three levels of IC on ToM. However, people in different individualistic and collectivistic regions are not only subject to different Country IC but they also internalize these cultural orientations differently (Individual IC), and are influenced by the cultural orientation of the immediate situation (Situational IC). This means that the three levels of analyses may reinforce each other. Therefore, we attempt to examine IC on the Country IC, Individual IC, and Situational IC levels and how they might influence ToM performance in combination.

### The present research

To test the above-mentioned hypotheses, for the Country IC level, we recruited participants from the Netherlands and Vietnam, representing individualism and collectivism, respectively. To assess the Individual IC, participants completed an individualism-collectivism questionnaire [36,55]. For the Situational IC level, participants were randomly assigned to one of three priming conditions: individualism-primed, collectivism-primed, and control (no-prime) conditions. The priming technique was based on the CSC framework: in a writing task, we asked participants to generate content that had a focus on either the individual “I” or the collective “we”. We measured ToM with the cartoon vignette task [23], which was chosen because it does not make any demands in terms of memory and language, which are frequently encountered as confounding factors when measuring ToM [56]. This task included trials that required ToM and trials that did not. We expected that greater accuracy and shorter reaction time associated with collectivism would only be present in the ToM trials and absent in the non-ToM trials (i.e. an interaction between cultural orientation and trial type). Our choice of task was intended to capture the differences in how participants, under different conditions of IC, and on different levels (country, individual, situation), put their ToM to use.

## Methods

### Participants

The study included a group of Dutch students and a group of Vietnamese students. One hundred and eighty-eight Dutch students at Vrije Universiteit Amsterdam (101 females, 86 males, one missing value), aged 16 to 33 (*M* = 22.28, *SD* = 4.455), were either recruited on campus or walked in the lab voluntarily. Twenty-two Dutch participants were excluded for different reasons: two guessed the research objective correctly, nine did not complete the priming task, six did not write the correct priming content, one was an outlier in terms of age, and the data of four participants were lost due to technical failure. The participant who was excluded because of age was 65 years old, while the remaining participants were between 16 and 30. Hultsch et al. [57] found that longer RTs in older adults (age 54–94), compared to younger adults (17–36), was due to cognitive functioning and aging, independently of experimental effects. Therefore, we removed this participant to exclude age as a confounding factor. Thirteen other participants were excluded from the analyses due to being outliers (i.e., 2*SD*s below and above the Dutch group mean): six participants were excluded for accuracy, five for reaction time and two for both accuracy and reaction time.

The Vietnamese sample consisted of 72 participants from 16 different universities in Ho Chi Minh city (38 females, 33 males, 1 missing value), with age ranging from 18 to 24 (*M* = 20.22, *SD* = 1.345). They received recruitment information from their lecturers at the National University of Social Sciences and Humanities and voluntarily participated by coming to the computer lab set up in an office rented by the researchers. The data of seven participants were excluded because they were outliers (i.e., 2*SD*s above and below the Vietnamese group mean): five participants were excluded for accuracy and two for reaction time. Another participant was excluded for not completing the Individual IC questionnaire. Out of the remaining 64 participants, ten were excluded because they did not write any priming content or this part of the data was lost due to a computer problem.

Both Dutch and Vietnamese participants were asked at the end of the experiment what they thought the tasks in the experiment were testing. We excluded participants who knew that the writing task was a priming task. This was because the effect of priming needed to be at an unconscious level [58]. It was possible that the participants would attempt to oppose the expected direction of the prime, a phenomenon known as the correction contrast [58]. The objective of the research was guessed correctly by two Dutch participants, but not by any Vietnamese participants. The exclusion of participants slightly affected the block assignment, especially in the Vietnamese sample. In the Dutch sample, there were 47 participants in the individualism-primed condition, 56 in the no-prime condition, and 50 in the collectivism-primed condition. In the Vietnamese sample, there were 16 participants in the individualism-primed condition, 21 in the no-prime condition, and 17 in the collectivism-primed condition. In summary, the final sample for the analyses consisted of 207 participants (n_Dutch_ = 153, n_Vietnamese_ = 54). The statistical analyses including the outliers can be found in S1 Text.

### Procedure and materials

The experiment was fully computerized and had a Dutch and a Vietnamese version. Instructions were translated and back-translated by the first author and research assistants. In the Netherlands, the experiment was run in the psychology laboratory of Vrije Universiteit Amsterdam, which had the capacity to simultaneously accommodate 36 participants in separate cubicles. In Vietnam, due to a lack of a standard psychology laboratory at local university campuses, a temporary two-cubicle laboratory, with two identically configured laptops, was set up in a leased office. Upon arriving at the labs, all participants were asked to give written consent prior to participation. The consent form provided them with information on the approximate duration, the number of tasks, and the general requirements of the tasks of the experiment (“you will write and read stories”). Participants were also informed that their participation was voluntary, that they could terminate it at any point without any consequences, and that their data would be treated with confidentiality. The study was checked and confirmed to be in line with the ethical guidelines of the Scientific and Ethical Review Committee of Vrije Universiteit Amsterdam, which, according to Dutch legislation, also sets the minimum age for providing consent at 16. Once they had given their consent, the participants were led to the cubicles and seated in front of a computer.

#### Priming individualism and collectivism (Situational IC)

First, participants were block-randomly assigned to one of the three individualism-primed, collectivism-primed, and no-prime/control conditions to ensure equal numbers of participants in each condition. Participants in the collectivism-primed and individualism-primed conditions were asked to “describe in a few (4–6) sentences an autobiographical situation in which you were with others/were alone and you made a joint decision/your own decision that had consequences for all of you/yourself”. Participants in the control (no-prime) condition went straight to the ToM task.

#### The cartoon vignette (the ToM task)

**Fig 1:**
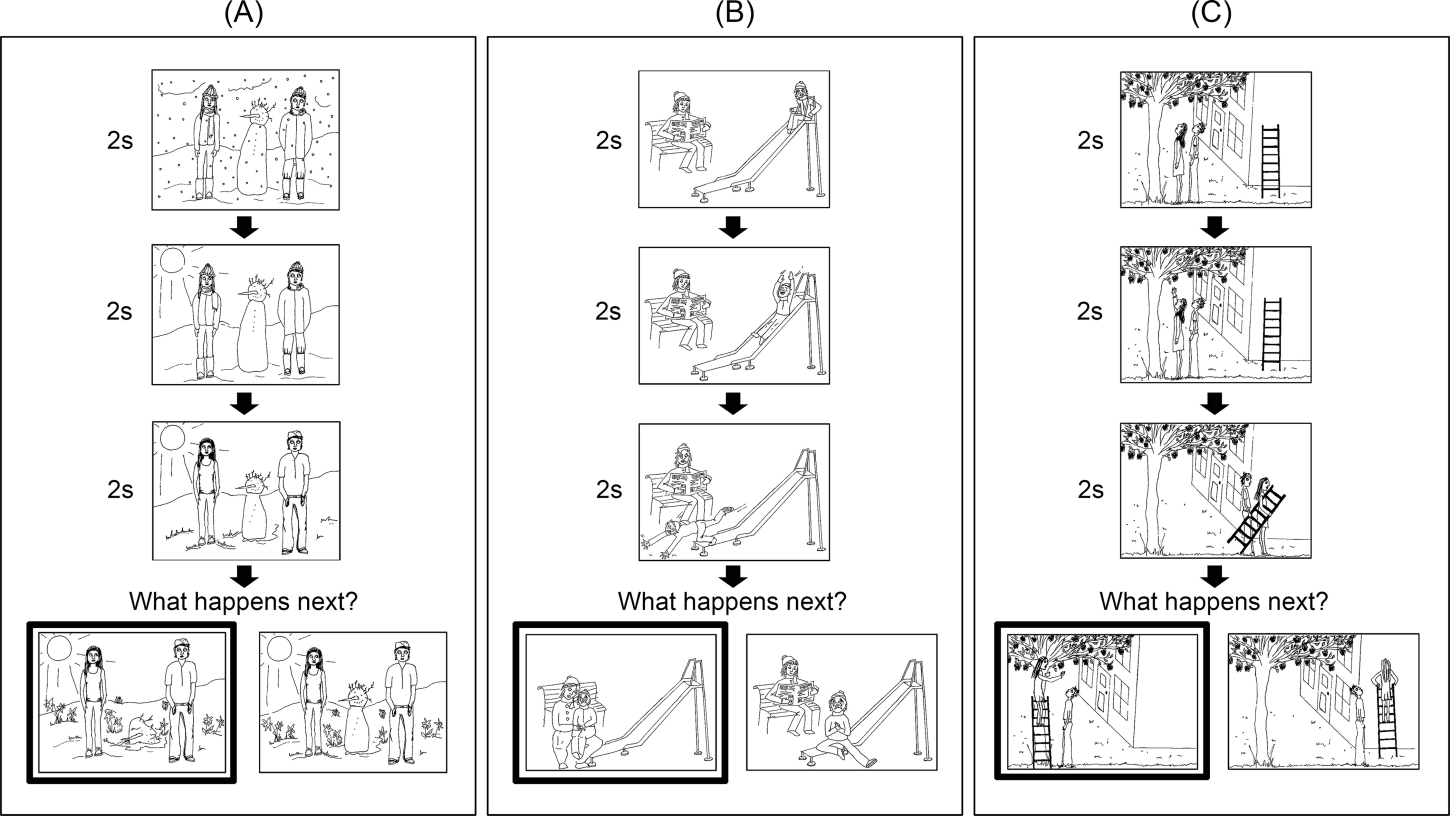
Examples of the cartoon vignette ToM task. (A) is a non-ToM, (B) an affective ToM, and (C) a cognitive ToM trial. The first three frames in each trial were presented back to back for 2000 ms each and then the choices (the last two frames) were presented simultaneously, from which the participants had to choose one or the other.

Participants’ ToM was then measured by the computerized cartoon vignette paradigm developed by Sebastian et al. [23], which allowed us to measure both accuracy and speed. Success in this task depended on participants’ understanding the social situations in these stories and the characters’ thoughts, feelings, and beliefs. The task consisted of 30 stories told in the form of comic strips. Ten stories required affective ToM, the ability to understand emotional states of the characters in the cartoons such as fear and pain. Ten stories required cognitive ToM, the ability to understand non-emotional mental states of the characters such as intention and motivation. Ten non-ToM stories required an understanding of physical causality, the ability to see cause-and-effect relationships between non-social objects (see Fig 1 for examples). Each story consisted of four frames depicting a simple social situation. Each trial began with a fixation cross presented for 200 milliseconds. Participants then saw the first three pictures in a sequence at 2-second intervals. Subsequently, participants were presented with two possible endings to the stories and the prompt, “What happens next?” The participants’ task was to choose, as quickly as possible, the picture that represented what they thought to be the most likely ending of each story. Participants made a binary decision by pressing either a left or right key on the keyboard. The location of the correct ending—either left or right—and the presentation order of the stories were randomly distributed. Prior to the 30 experimental trials, participants had three practice trials that included more elaborate step-by-step instructions than those in the experimental trials. However, no feedback was given in the practice or experimental trials.

#### Individualism-collectivism measure (Individual IC)

Participants were subsequently asked to complete a shortened 10-item version of the Individualism–Collectivism Measure [36,55], which assessed their individual IC. Five of the items were collectivism items (e.g., “My happiness is dependent on the well-being of my close others”) and five were individualism items (e.g., “It is important to be myself, despite what other people think”). The scale ranged from 1 (*strongly disagree*) to 7 (*strongly agree*). In the Dutch sample, the reliability of the collectivism measure was Cronbach’s *α* = .705 and that of the individualism counterpart Cronbach’s *α* = .758. In the Vietnamese sample, they were *α* = .583 and *α* = .653, respectively. For each participant, we calculated mean scores for collectivism and individualism and established a single individualism-collectivism ratio (ranging from -.86 to 1.45) by subtracting each participant’s mean individualism score from their mean collectivism score and then dividing the result by their own average individualism score. A higher ratio represented predominantly collectivistic values. Since our hypothesis predicted a positive relationship between collectivism and ToM, it was natural to choose a ratio that reflected the degree of collectivism that took into account within-participant individualism. In the main analyses, a mean-centered version of this ratio was used to ensure meaningful coefficient values [59].

Finally, the participants answered questions about their age, gender, school, study major, study year, ethnicity, and region. Before leaving the cubicle, they were fully debriefed about the purpose of the experiment and the deception in the writing task. The participants were then given the opportunity to withdraw from participation or submit their data. Regardless of their choice, participants received compensation of either course credit or EUR 2.5 or VND 50,000. Before leaving, the participants were asked to provide their student or social security numbers on a separate form that was not connected to any part of the data collected in the cubicles, in order to obtain their study credit or financial reimbursement. We later sent the study credits and social security numbers of the Dutch participants to the independent educational administration and the financial control department of Vrije Universiteit Amsterdam.

There were important differences between the Dutch participants’ experiences and those of their Vietnamese counterparts. Firstly, the Dutch participants were immediately assigned to a cubicle; whereas, due to the limited number of cubicles, several Vietnamese participants waited for half an hour and a few extreme cases waited for two hours. Secondly, the majority of the Dutch participants had routinely taken part in psychological experiments, while all of the Vietnamese participants reported verbally to the research assistant that it was the first time they had participated in such an experiment. Thirdly, the Dutch participants spent about 15–30 minutes completing the experiment, while the Vietnamese participants spent about 45–60 minutes. Finally, when they learned that the study not only required filling out questionnaires but also engaging in a computerized task, the Vietnamese participants expressed considerable anxiety and asked if they could have more time. None of the Dutch participants expressed a similar concern. In summary, there were considerable group differences in the experience in the lab.

## Results

### Manipulation check

Two native speakers of Dutch and two native speakers of Vietnamese who were blind to the nature of the study independently content-coded the autobiographical stories that the participants supplied in the priming task. For most participants, the writing content corresponded to the priming condition to which the participants were assigned. As noted above, the participants who did not write about the priming content in line with the requirements were excluded from the final sample. In summary, the manipulation succeeded in providing cues for collectivistic and individualistic mindsets in the participants, whose data were further used in the analyses.

### Individualism-collectivism measure

The Dutch participants (*M* = 4.82, *SD* = .95) were significantly more individualistic than the Vietnamese participants (*M* = 4.43, *SD* = 1.01, *t*(215) = 1.71, *p* = .007, 95% CI [.11, .67]). The Dutch participants (*M* = 4.73, *SD* = .87) were also significantly less collectivistic than the Vietnamese participants (*M* = 5.03, *SD* = .82, *t*(215) = -2.25, *p* = .025, 95% CI [-.55, -.04]). This is in line with previous research, which also generally found that people from a Western European cultural heritage score higher on individualism and lower on collectivism than people of East Asian cultural heritage [55].

### Country IC, Individual IC, and Situational IC analyses

Initially, we intended to perform a 3 x 2 x 3 Mixed Design Repeated Measures ANCOVA with Trial Type (affective ToM, cognitive ToM, and non-ToM trials), Country IC (Dutch and Vietnamese), Situational IC (individualism-primed, collectivism-primed, and no-prime conditions), and Individual IC (the mean-centered individualism-collectivism ratio). The statistical strategy was to simultaneously test the three-level effects of culture on ToM performance. However, descriptive statistics on reaction time in the ToM task called for special attention.

Following the steps taken by the original authors of the task [23], we only included the reaction times in correct trials when calculating average reaction time for each participant. On average, the Dutch participants needed 1902.681 ms (*SD* = 66.221) to complete a trial, while the Vietnamese participants needed 4061.066 ms (*SD* = 116.065). Such a difference (*t*(215) = -18.512, *p* < .001, 95% CI [-2563.135; -2069.829]) between the two groups seemed too large to be solely attributable to any experimental effect. Instead, the above-mentioned unanticipated cultural interaction with the experimental conditions might have contributed to this difference.

Given these findings and considerations, we decided to analyze the data of the Dutch and Vietnamese participants separately. The two-level Country IC predictor was removed from the initially planned Mixed Design Repeated Measures ANCOVA. The final statistical approach was thus a 3 x 3 Mixed Design Repeated Measures ANCOVA with Trial Type (affective ToM, cognitive ToM, and non-ToM), Situational IC (individualism-primed, collectivism-primed, and no-prime conditions) and Individual IC. For each group, two such ANCOVAs were conducted: one on ToM accuracy (affective ToM, cognitive ToM, and non-ToM accuracy), and the other on reaction time (affective ToM, cognitive ToM, and non-ToM reaction time).

#### Accuracy in the Dutch sample (see Fig 2 and S1A Table)

Greenhouse-Geisser correction was used to correct for non-sphericity. In the Dutch sample, there were no effects of Individual IC (*F*(1, 147) = 1.173, *p* = .280) or of Situational IC (*F*(2, 147) = 0.630, *p* = .534) on the number of correct answers in the ToM task (ToM accuracy). The two-way interaction between Individual IC and Situational IC was also non-significant (*F*(2, 147) = 2.134, *p* = .122). The main effect of Trial Type on accuracy was, however, significant (*F*(1.674, 246.068) = 17.650, *p* < .001, *η*^*2*^ = .107). Dutch participants were least accurate in affective ToM. The difference between affective ToM (*M* = 8.839, *SE* = .102) and cognitive ToM (*M* = 9.437, *SE* = .070) was significant (*F*(1,147) = 27.034, *p* < .001). The difference between cognitive ToM and non-ToM (*M* = 9.261, *SE* = .067) was also significant (*F*(1,147) = 5.460, *p* = .021). The two-way interaction between Trial Type and Individual IC was non-significant (*F*(1.674, 246.068) = 0.538, *p* = .553), indicating that the accuracy differences between types of trials were not affected by the individualism-collectivism scores. The two-way interaction between Trial Type and Situational IC was also non-significant (*F*(3.348, 246.068) = 0.091, *p* = .974). This means that no matter which priming condition (Situational IC) they were in, Dutch participants were more accurate at making inferences about the thoughts of others than they were at assessing the feelings of the targets.

**Fig 2:**
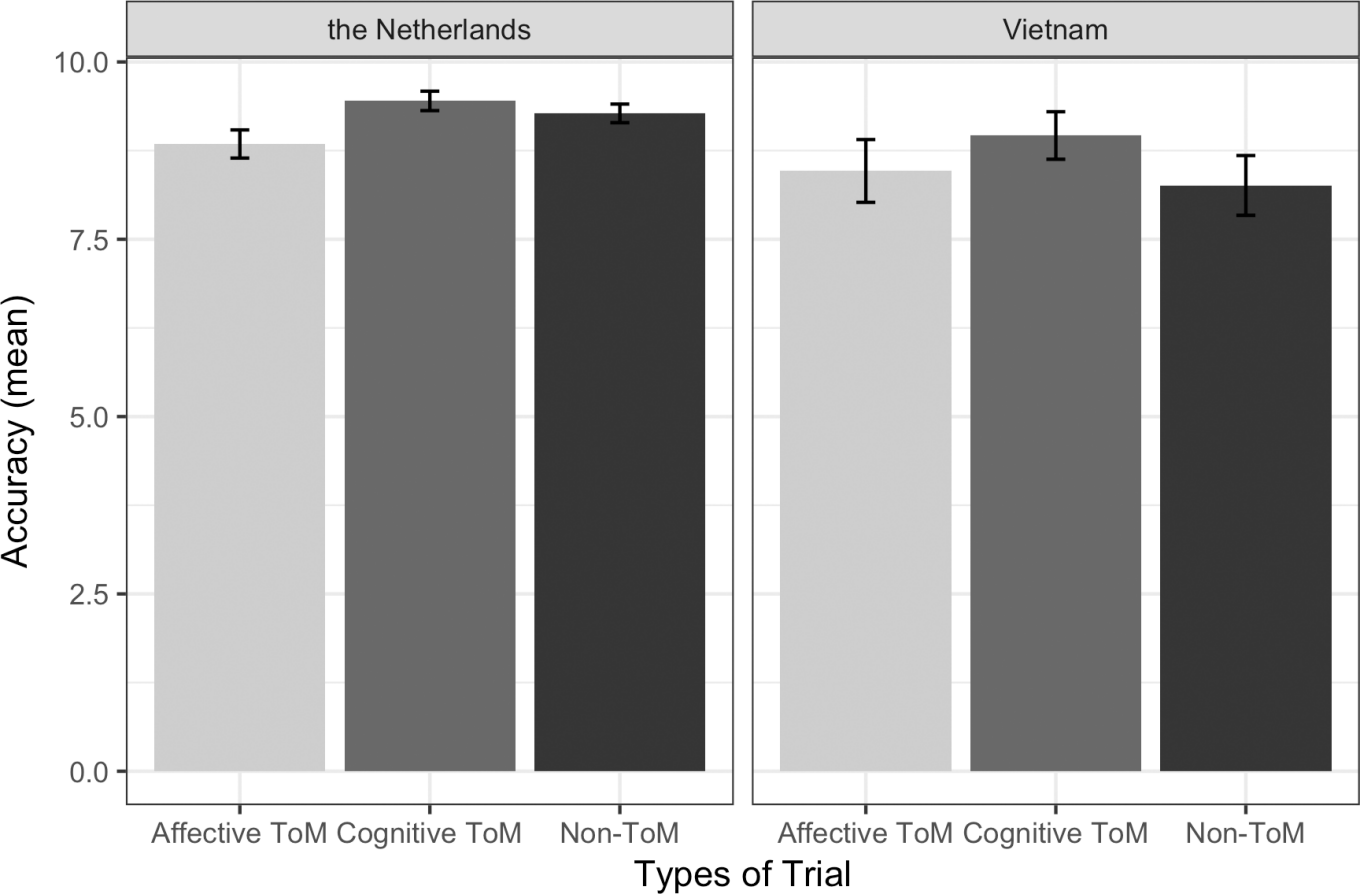
ToM accuracy. Average correct objects split by Trial Type and Country IC. Error bars are the standard errors of the means.

#### Accuracy in the Vietnamese sample (see Fig 2 and S1A Table)

**Fig 3:**
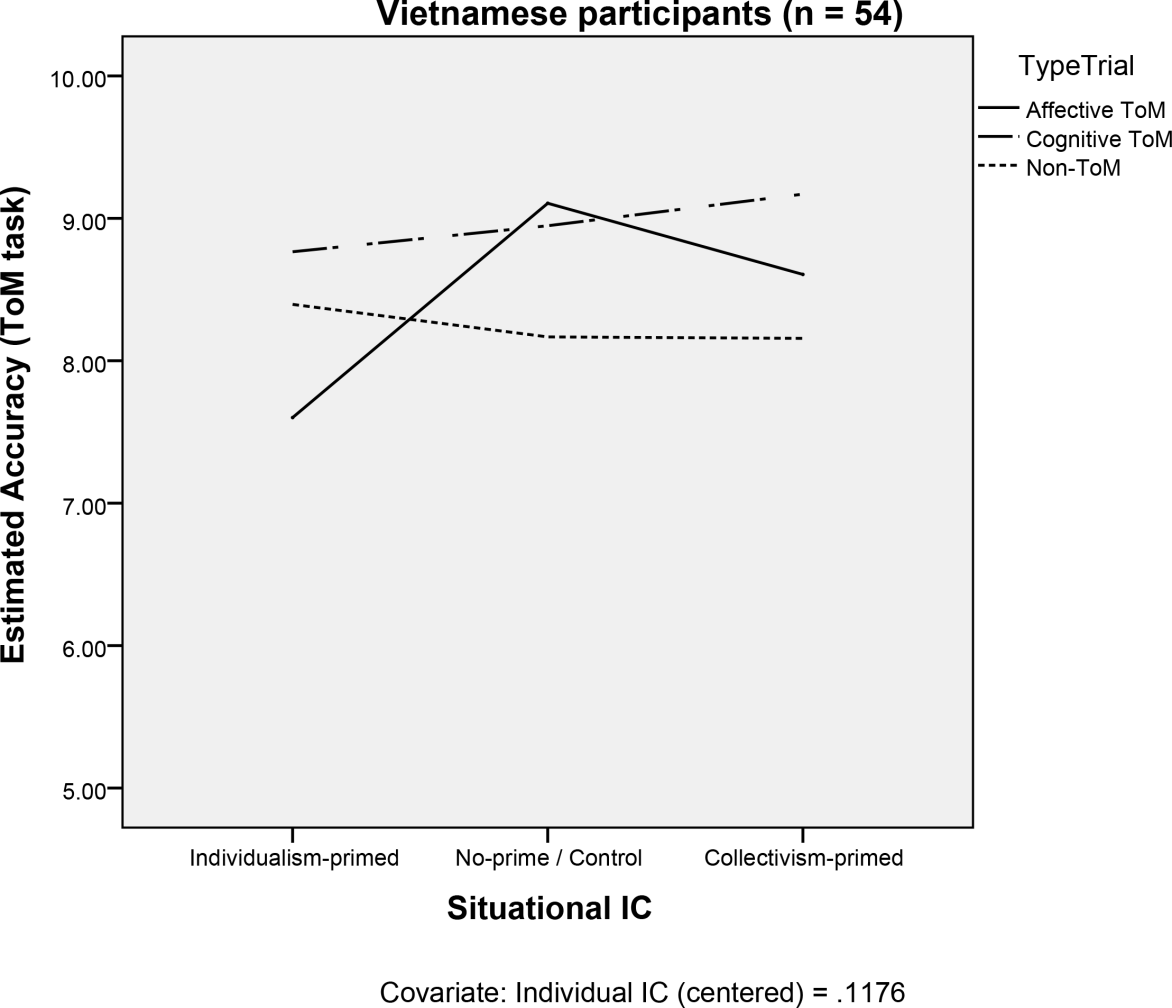
The Situational IC and Type Trial interaction in the Vietnamese sample. The individualism-primed participants were significantly less accurate than the collectivism-primed participants at affective ToM while there were no differences between the primed groups in cognitive ToM and non-ToM trials.

In the Vietnamese sample, there were no effects of Individual IC (*F*(1, 48) = 2.979, *p* = .091) or of Situational IC (*F*(2, 48) = 1.737, *p* = .187) on ToM accuracy. The two-way interaction between Individual IC and Situational IC was non-significant (*F*(2, 48) = 0.862, *p* = .429). The main effect of Trial Type was marginal (*F*(2, 96) = 3.053, *p* = .052). However, the two-way interaction between Trial Type and Situational IC was significant (*F*(4, 96) = 2.957, *p* = .024). There were differences in terms of accuracy between types of trials, but this was conditional on priming conditions (Situational IC) (see Fig 3). Specifically, with regard to evaluating feelings (affective ToM trials), the individualism-primed Vietnamese participants were significantly less accurate than the collectivism-primed participants (*B* = -1.334, *t*(48) = -2.670, *p* = .010, 95% CI [-2.338; -.329]). The two-way interaction between Trial Type and Individual IC was non-significant (*F*(2, 96) = 1.439, *p* = .242).

#### Reaction time in the Dutch sample (see Fig 4 and S1B Table)

In the Dutch sample, there were no effects of Individual IC (*F*(1, 147) = 0.653, *p* = .420) or of Situational IC (*F*(2, 147) = 2.234, *p* = .111) on how fast participants responded in the ToM task (ToM reaction time). The two-way interaction between Individual IC and Situational IC was non-significant (*F*(2, 147) = 0.120, *p* = .887). The main effect of Trial Type on reaction time was also non-significant (*F*(2, 294) = 2.735, *p* = .067, *η*^*2*^ = .018). The Dutch participants were equally fast, regardless of whether they were evaluating the feelings (affective ToM trials) or the thoughts (cognitive ToM trials) of the characters in the stories, or the physical causality between non-social objects (non-ToM trials).

**Fig 4:**
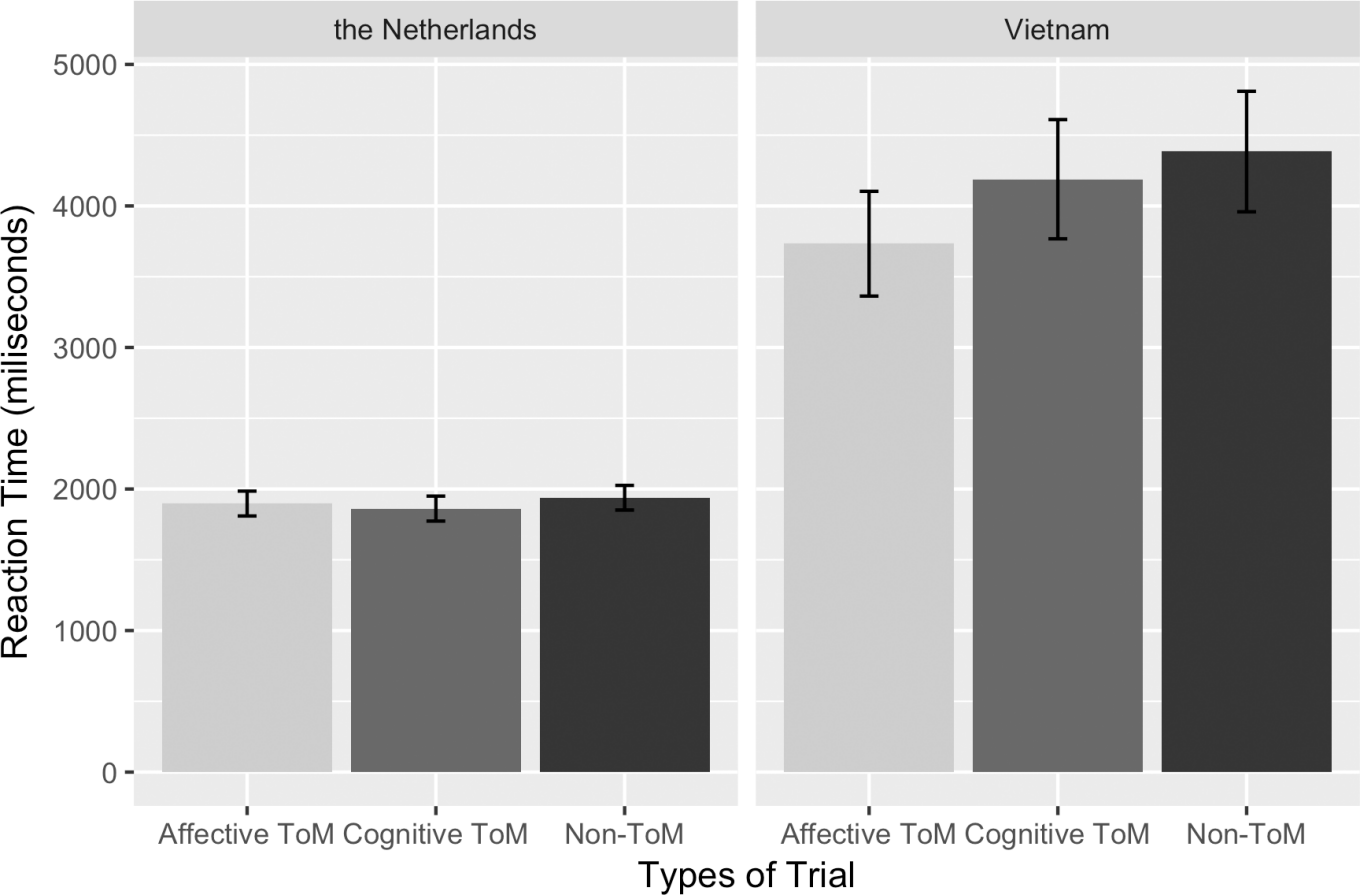
ToM reaction time. Average response time participants needed to reach the correct objects split by Trial Type and Country IC. Error bars are the standard errors of the means.

#### Reaction time in the Vietnamese sample (see Fig 4 and S1B Table)

Greenhouse-Geisser correction was used to correct for non-sphericity. In the Vietnamese sample, there were no effects of Individual IC (*F*(1, 48) = 0.001, *p* = .972) or of Situational IC (*F*(2, 48) = 0.039, *p* = .962) on reaction time in the ToM task. The two-way interaction between Individual IC and Situational IC was non-significant (*F*(2, 48) = 1.751, *p* = .185). The main effect of Trial Type on reaction time was significant (*F*(1.596, 76.592) = 4.836, *p* = .016, *η*^*2*^ = .092). Vietnamese participants were faster when evaluating the feelings of the characters (affective ToM trials, *M* = 3693.236, *SE* = 195.992) than when judging the thoughts of the characters (cognitive ToM trials, *M* = 4084.200, *SE* = 215.370, *F*(1,48) = 6.826, *p* = .012). They were equally slow when judging thoughts (cognitive ToM) and when judging physical causality (non-ToM trials) (*M* = 4287.845, *SE* = 215.823, *F*(1,48) = .862, *p* = .358). The lack of a two-way interaction between Trial Type and Individual IC (*F*(1.596, 76.592) = 1.163, *p* = .309) and between Trial Type and Situational IC (*F*(3.191, 76.592) = 0.714, *p* = .555) indicated that these differences between types of trial remained unchanged across Situational IC conditions and did not depend on Individual IC either.

## Discussion

The present research investigated whether individualism and collectivism on three different levels (country, individual, and situation) influenced how well a person could make inferences about the thoughts and feelings of another person (i.e. ToM performance). A stark contrast between the Dutch and the Vietnamese participants observed during data collection, and the large differences in their reaction times led to the decision to analyze the data separately for each group. Consequently, we were unable to evaluate the effect of individualism and collectivism on ToM performance at the country level (Country IC). The group-wise analyses further suggested that Individual IC did not have an impact on ToM performance. Situational IC had some influence on ToM performance but the effect was only observed in the collectivist Country IC group and was dependent on the type of ToM. Below, we discuss our findings.

### The effect of Country IC on ToM and the implications for experimental cross-cultural research

From our data, it is not possible to conclude whether living in a predominantly collectivistic country, compared to living in a predominantly individualistic country, is associated with more efficient ToM. We suggest that the large difference in RT between the Dutch and Vietnamese participants might not be attributable to the experimental manipulation and could be partially traced back to an unanticipated interaction between the participants’ cultural differences and the experimental settings. Firstly, participating in experiments was new and daunting for Vietnamese participants, which could have triggered anxiety among them, but this did not occur among Dutch participants. Recent research has indeed found that anxiety impairs the spontaneous tendency to take another person’s perspective [60]. Secondly, inexperience with computerized tasks could have resulted in longer RTs generally in Vietnamese participants. Thirdly, Vietnamese participants were willing to wait, while no Dutch participants had to or would be willing to do so. This contrast in motivation may have led to excessive prudence among Vietnamese participants. Finally, Vietnamese participants could have a different relationship to time than Dutch participants did, given that the perception of time in collectivistic countries is slower than in individualistic countries [61]. In combination, anxiety, excessive prudence, and slower time perception could have influenced how the Vietnamese participants handled the tasks and led to longer reaction times.

The potential interaction between cultural differences and the experimental setting may not have attracted much attention in earlier research for two reasons. Firstly, the majority of previous Western versus non-Western studies were not physically carried out across Western and non-Western countries. In these studies, the non-Western participants were often not residing in their home countries. Instead, most experiments were conducted in Western Europe or North America, where the collectivism-representing participants lived and studied [9,14,39,43,46,62]. Secondly, even when cross-country replications are carried out, identical experimental paradigms are often not used. These two features hold true for most previous research that found a link between collectivism and efficiency in ToM.

However, the lack of true cross-cultural comparison may be problematic. The fact that participants in the previous studies were removed from their native geographical and cultural contexts, which guide their cognition and behaviors, necessitates caution in attributing the difference in their performance solely to cultural differences. People who live in their country of origin may differ from those who emigrated (or whose parents did) in a large variety of ways [54], and future research might examine which are most influential.

It has also been argued that cross-cultural research may benefit from internationalization efforts, which include reaching out to diverse samples and conducting research in different countries [63]. However, researchers who are involved in internationalized research may stumble upon new challenges, one of which, as we discovered, is how to ensure the internal validity of the experimental paradigm. Our recommendation for future research is to pursue a true cross-cultural approach and to simultaneously implement certain measures to obtain more comparable experimental outcomes across countries.

Based on the guideline developed by Van de Vijver [63], we can list a few suggestions on how to identify biases that are inherent in inferences across cultures and that need to be taken into account. Firstly, a construct may not correspond to differences in the same underlying trait (e.g., RTs in the Netherlands may be a consequence of competitiveness and the importance of personal achievement and therefore tend to be short, while they may be a consequence of a sense of social duty in Vietnam and therefore long). Researchers could combine different measures (e.g., self-reports of ToM in addition to RTs in ToM performance in a computerized task) and attempt to combine the results across different measures to reach a more informative conclusion. Secondly, bias may result from methodological aspects, including the sample, the instruments, and the administration. The samples may not be comparable. For example, as discussed above, due to their cultural background, participants might interact differently with the experimental conditions. Thus, bias from the instruments may be due to the fact that people in two countries differ in their level of familiarity with stimuli (or, in our case, with participating in experimental research at all), and in response styles (e.g., perception of time). A remedy for this could be that participants inexperienced with computerized tasks be given preparatory tasks prior to the main experimental task. Finally, bias may result from differences in administration conditions (e.g., lab facilities, waiting time, etc.). These factors may have contributed to the differences we found between the studies in the two countries. For this reason, the minute details regarding the facilities in the labs, such as the use of time and the pace of operation, should also be comparable, especially when the effect is expected to be captured by reaction time.

### The effect of Individual IC and Situational IC on ToM

Using the IC questionnaire, we were able to investigate the relationship between individualism and collectivism at the individual level (Individual IC) and ToM. In contrast to our hypothesis, in neither the Dutch nor the Vietnamese group was the degree of collectivism associated with accuracy or speed of evaluating the thoughts and feelings of another person. We only found that Situational IC interacted with the type of ToM in the Vietnamese sample: being placed in a situation that cued individualism, compared to being placed in a situation that cued collectivism, resulted in less accurate understanding of another person’s feelings (affective ToM).

There are two possible explanations for the absence of main effects of Individual IC and Situational IC on ToM. The first possibility is that the efficiency associated with individualism unexpectedly cancelled out that of collectivism. An important characteristic of individualism is an inclination for competition and personal achievement [33]. While collectivism might lead to more accurate and faster ToM performance because participants are more sensitive to the thoughts and feelings of others, individualism could also lead to faster performance simply because participants want to excel and demonstrate their mastery in any task.

The second possibility is that the hypothesized effect of IC on ToM was not captured by our dependent measure task. This might be because it did not distinctively target the type of mental states related to I or C. For example, affective ToM trials only tested whether participants could recognize the need to generate an overt empathic response to those who were in fear or pain (e.g., the child who falls from a slide needs verbal comfort). The overt display of empathy might not be particularly tied to collectivism because the open display of emotion is not what people from a dominantly collectivistic culture are accustomed to [64]. Atkins et al. [22] actually found that, despite more accurately understanding another’s feelings, their collectivism-representing Chinese participants did not display their empathy more strongly than the individualism-representing British counterparts.

Similarly, our cognitive ToM trials heavily focused on understanding a single mental state: intention with regard to physical objects (e.g., the person who grabs a ladder wants to pick fruit from a tree). It is more likely that tracking intentions with regard to physical objects is within the field of influence of individualism rather than collectivism.

Furthermore, collectivism could be specifically related to the kind of ToM that enables people to guarantee harmony and group cohesion because this is where collectivism and individualism differ most prominently. For example, it might be highly relevant for those from a C society, or with a C personality, or in a C situation to be more accurate and faster to detect if someone is angry because group harmony might be in danger. Likewise, given the requirements of a C society, or C personality, or a C situation to understand what someone wants to do for another person (which requires affective ToM) may be more relevant than understanding what someone wants to do with an object (which requires cognitive ToM). Future research should choose a task that covers the mental states in which participants’ vigilance with respect to a disruption or threat to harmony and group cohesion can be specifically targeted.

Finally, the ToM cartoon task we used might not be optimal to measure differences in ToM performance because it only required participants to make passive inferences about the mental states of others, without prompting them to act on the information garnered. In contrast, in Wu and Keysar’s study [9], where they found collectivism to be associated with more efficient perspective-taking than individualism, the task required participants to cooperate with the target person in an ongoing interaction. This could also be where collectivism manifests an effect on ToM: a situation in which participants have an interdependent relationship with the target person, whose mental states they need to infer. In addition, their dependent measure was eye gaze rather than accuracy and RT; thus, it is possible that the effect that collectivism may have on ToM might be more sensitive to eye gaze measures.

### The main effect of types of ToM and their interaction with IC

Although we did not find evidence for any effect of IC on ToM performance, an interesting pattern emerged in the accuracy and reaction time performances of the Dutch and Vietnamese participants. Regardless of the priming conditions, Dutch participants were least accurate in affective ToM. In contrast, when primed with individualism, the performance of Vietnamese participants specifically in affective ToM declined, compared to when they were primed with collectivism or assigned to the control condition. Similar patterns were observed in the reaction time data. Whether evaluating feelings, thoughts, or physical causality, Dutch participants were equally fast. In contrast, Vietnamese participants were actually fastest at evaluating the feelings of others and were slowest at evaluating the thoughts of others and physical causality.

These findings suggest a tentative post-hoc hypothesis that warrants future research: cognitive ToM and affective ToM might be linked to individualism and collectivism, respectively. Neuropsychological and brain-imaging studies have revealed that cognitive mental states (beliefs, desires, intentions, etc.) and affective mental states (feelings and emotions), despite some overlap, have distinguishable neural representations [23,65]. A possible mechanism is that a small overlap between the representation of the self and of others might be conducive to cognitive ToM because it requires differentiating one’s thoughts from those of others. In contrast, affective ToM might be facilitated by a large self-other overlap because it may be enabled by the simulation of other people’s feelings. Living in a collectivistic country, Vietnamese participants may, on average, have a larger self-other overlap, while Dutch participants, living in an individualistic country may, on average, have a smaller self-other overlap [66]. This difference might underlie why Dutch participants did not do particularly well in affective ToM, while the Vietnamese participants were best in affective ToM.

Looking back at previous research, it also seems that the ToM-related processes, on which collectivism was found to have a boosting impact, were more similar to affective ToM than to cognitive ToM. Cohen and Gunz [39] found an effect of collectivism on tasks in which participants were asked to recall affect-laden memories. Atkins et al. [22] and Duan et al. [43] found an effect of collectivism on empathy accuracy and empathy concern, which are more similar to affective ToM than to cognitive ToM. An exception are the results of Wu and Keysar [9], who found an effect of collectivism on perspective-taking, which can be classified as cognitive ToM. However, as discussed above, the independent nature of the task and the use of eye gaze as the dependent measure may actually make the task more sensitive to cultural influences in general.

Regarding Vietnamese participants, on whom the priming effect was manifest to a certain extent, being in an individualistic situation, or having to think of themselves as an independent entity separate from others, was detrimental to their accuracy performance. This finding is in line with the cultural fluency model [67], which posits that culturally congruent situations are conducive to the task at hand, while culturally incongruent situations may hinder task performance. In this vein, the individualism-primed Vietnamese participants, who lived in a collectivistic society, were in a culturally incongruent situation and their ToM performance declined. This explanation, however, fails to account for one non-finding: the lack of a priming effect on the Dutch participants’ accuracy performance (as opposed to the presence of such interaction in the Vietnamese sample). It is possible that the priming effect of individualism and collectivism might not be robust.

Finally, apart from the 72 Vietnamese participants, eight other Vietnamese students came to the lab but decided not to participate in the experiment. Perhaps some of the potential participants who arrived at the time when there was a queue in the lab were dissuaded from taking part in the experiment. If so, this might have created a selection bias towards those who were most patient or willing to make sacrifices for the group. However, those who had to wait were a minority and specific reasons for why those who came but eventually left without participating seemed to vary. Therefore, it seems unlikely that such a selection bias was introduced in the data to the extent that patience and dedication drove the effect in the Vietnamese sample.

## Concluding remarks

The juxtaposition of the absence of main effects (Individual IC and Situational IC) and the presence of the interaction between Situational IC and the type of ToM necessitates a review of the hypothesis that we initially proposed. In relation to this hypothesis, we did not find evidence that IC on any level has an effect on ToM, or, if such an effect exists in the population, it might not necessarily be captured in the ToM task that we used. However, we found some indication that individualism and collectivism could have boosting influences, not on general ToM, but on different types of ToM. Despite several limitations, the present research still offers several contributions to the research in the field. Our study is among only a few that attempt to use multilevel analyses (country, individual, and situational level) of culture in cross-cultural research. Furthermore, our experience in using diverse and hard-to-reach participant pools highlights issues in experimental cross-cultural research, which will be beneficial for future research.

## Supporting information

S1 Table Overview of the mixed design repeated measures ANCOVA on ToM accuracy and on ToM reaction time. (DOCX)

S1 Text Statistical analyses including outliers. (DOCX)
