# Supplementary material for: Do individualism and collectivism on three levels (country, individual, and situation) influence theory-of-mind efficiency? A cross-country study
Source: PLoS One. 2017 Aug 23;12(8):e0183011. doi: 10.1371/journal.pone.0183011 (PMC5568381; doi:10.1371/journal.pone.0183011)
Supplement: S1 Table — (DOCX) [file pone.0183011.s001.docx]

|  | | **Dutch** (n = 153) | | | | | **Vietnamese** (n = 54) | | |
| --- | --- | --- | --- | --- | --- | --- | --- | --- | --- |
| **Predictors** | df | | Error | *F* | *p* | df | Error | *F* | p |
| **Individual IC** | 1 | | 147 | 1.173 | .280 | 1 | 48 | 2.979 | .091 |
| **Situational IC** | 2 | | 147 | 0.630 | .534 | 2 | 48 | 1.737 | .187 |
| **Individual IC** x **Situational IC** | 2 | | 147 | 2.134 | .122 | 2 | 48 | 0.862 | .429 |
| **Trial Type** | 1.674 | | 246.068 | 17.650 | <. 001 | 2 | 96 | 3.053 | .052 |
| **Trial Type** x **Individual IC** | 1.674 | | 246.068 | 0.538 | .553 | 2 | 96 | 1.439 | .242 |
| **Trial Type** x **Situational IC** | 3.348 | | 246.068 | 0.091 | .974 | 4 | 96 | 2.957 | .024 |

**S1 Table A. Results of the mixed design repeated measures ANCOVA on ToM accuracy, shown separately for the Dutch & Vietnamese groups.**

df = degree of freedom. Greenhouse-Geisser correction was used to correct for non-sphericity in the Dutch sample.

**S1 Table B. Results of the mixed design repeated measures ANCOVA on ToM reaction time, shown separately for the Dutch & Vietnamese groups.**

|  | | **Dutch** (n = 153) | | | | | **Vietnamese** (n = 54) | | |
| --- | --- | --- | --- | --- | --- | --- | --- | --- | --- |
| Predictors | df | | Error | *F* | *p* | df | Error | *F* | p |
| **Individual IC** | 1 | | 147 | 0.653 | .420 | 1 | 48 | 0.001 | .972 |
| **Situational IC** | 2 | | 147 | 2.234 | .111 | 2 | 48 | 0.039 | .962 |
| **Individual IC** x **Situational IC** | 2 | | 147 | 0.120 | .887 | 2 | 48 | 1.751 | .185 |
| **Trial Type** | 2 | | 294 | 2.735 | .067 | 1.596 | 76.592 | 4.836 | .016 |
| **Trial Type** x **Individual IC** | 2 | | 294 | 1.277 | .280 | 1.596 | 76.592 | 1.163 | .309 |
| **Trial Type** x **Situational IC** | 4 | | 294 | 0.600 | .663 | 3.191 | 76.592 | 0.714 | .555 |

df = degree of freedom. Greenhouse-Geisser correction was used to correct for non-sphericity in the Vietnamese sample
