# Supplementary material for: Do individualism and collectivism on three levels (country, individual, and situation) influence theory-of-mind efficiency? A cross-country study
Source: PLoS One. 2017 Aug 23;12(8):e0183011. doi: 10.1371/journal.pone.0183011 (PMC5568381; doi:10.1371/journal.pone.0183011)
Supplement: S1 Text — (DOCX) [file pone.0183011.s002.docx]

**S1 Text. Statistical analyses including outliers**

Dutch sample

Thirteen other participants were excluded from the analyses due to being outliers (2*SD*s below and above the Dutch group mean): six in terms of accuracy, five in reaction time and two in both accuracy and reaction time.

When the outliers in terms of accuracy and RT (2SD away from the group mean) were included in the analysis, most results did not deviate from what we had found except for this main effect of Type Trial, which reached significance (*F*(2,108) = 4.207, *p* = .017, *η^2^* = .072). Vietnamese participants were comparably accurate at Affective ToM (*M* = 8.205, *SE* = .211) and Cognitive ToM (*M* = 8.673, *SE* = .209), *F*(1,54) = 3.694, *p* = .060. However, they were significantly less accurate at non-ToM (*M* = 7.792, *SE* = .285), compared to Cognitive ToM, *F*(1,54) = 7.738, *p* = .007. Nevertheless, since there was an interaction between Trial Type and Situational IC, the main effect of Trial Type was not interpreted.

Vietnamese sample

Data of seven participants were excluded because of being outliers (also 2*SD*s above and below the Vietnamese group mean, five in terms of accuracy and two in reaction time).

When the outliers in terms of accuracy and RT (2SD away from the group mean) were included in the analysis, most results did not deviate from what we had found except for this main effect of Type Trial, which reached significance (*F*(2,108) = 4.207, *p* = .017, *η^2^* = .072). Vietnamese participants were comparably accurate at Affective ToM (*M* = 8.205, *SE* = .211) and Cognitive ToM (*M* = 8.673, *SE* = .209), *F*(1,54) = 3.694, *p* = .060. However, they were significantly less accurate at non-ToM (*M* = 7.792, *SE* = .285), compared to Cognitive ToM, *F*(1,54) = 7.738, *p* = .007. Nevertheless, since there was an interaction between Trial Type and Situational IC, the main effect of Trial Type was not interpreted.
